# Use of physiological signals, behavioral data, and processing algorithms in electronic devices and mobile applications for diagnosing depression, anxiety, and stress

**DOI:** 10.1177/20552076251404514

**Published:** 2026-01-19

**Authors:** Camila Alexandra Castillo Zorro, Gregory A Fonzo, William D Moscoso-Barrera

**Affiliations:** 1 Faculty of Engineering and Basic Sciences, 27979 Universidad Central, Bogotá, Colombia; 2 Center for Psychedelic Research and Therapy, Department of Psychiatry and Behavioral Sciences, 377659 Dell Medical School, The University of Texas at Austin, Austin, TX, USA; 3 Department of Biomedical Engineering, Cockrell School of Engineering, 12330 The University of Texas at Austin, Austin, TX, USA

**Keywords:** Depression, anxiety, stress, electronic devices, mobile applications, physiological signals, behavioral data, diagnosis, processing algorithms

## Abstract

**Objective:**

To review the technologies, biomarkers and processing algorithms used in diagnosing depression, anxiety, and stress, informing future research endeavors to enhance healthcare and the well-being of individuals affected by these mental disorders.

**Methods:**

A systematic review was conducted through searches in electronic databases such as PubMed, Google Scholar, IEEE, Nature, ProQuest, and Science Direct. Search queries combined terms related to physiological signals, behavioral variables, electronic devices, mobile applications, and the disorders of depression, anxiety, and stress. After screening 292 initial records, 77 studies met specific criteria, which included discussion of quantitative results and advanced processing algorithms.

**Results:**

The review of 77 articles revealed an increasing use of electronic devices and applications for measuring physiological and behavioral variables in the diagnosis of mental disorders. The wrist was the most common device location, accounting for 53.2%, primarily utilizing smartwatches to monitor heart rate, electrodermal activity, and sleep patterns. The analyzed technologies included wearable sensors, facial recognition cameras, electroencephalographs, and virtual reality devices. The classification algorithms used—such as decision trees, neural networks, and support vector machines—achieved accuracy rates ranging from 75% to 90%, highlighting the effectiveness of these tools. However, limitations were identified regarding the generalizability of results and the need for personalized diagnostic models.

**Conclusion:**

Electronic devices and mobile applications represent a significant advancement in the detection and monitoring of depression, anxiety, and stress by providing objective data continuously and in real time. However, their clinical application still faces challenges related to accuracy, personalization, and user acceptance. For these technologies to be effectively integrated into clinical practice, it is recommended to conduct studies in real-world settings and foster collaboration with mental health professionals. Such efforts would enable the adaptation of diagnostic models to individual needs and enhance the accuracy of early interventions.

## Introduction

Mental disorders impact an individual's thinking, emotions, mood, and behavior, affecting the ability to perform daily activities and maintain social relationships. In 2019, approximately 970 million people worldwide were affected by some form of mental disorder.^1^ Among the most common mental health disorders are anxiety and depression. According to the World Health Organization (WHO), it is estimated that over 280 million people globally suffer from depression, representing approximately 3.8% of the global population.^2^ Furthermore, it is reported that 301 million people live with some form of anxiety disorder.^3^

Depression is a complex mental disorder that affects a large number of individuals globally, with various contributing factors, including biological, psychological, and social influences.^4^ Characteristic symptoms of depression include persistent sadness, loss of interest or pleasure in previously enjoyed activities, changes in appetite and weight, difficulty concentrating, feelings of guilt or worthlessness, low energy levels, and thoughts of death or suicide.^5^ Similarly, anxiety is a common condition that affects numerous people worldwide, presenting itself through panic attacks, phobias or generalized anxiety disorder. Multiple factors contribute to the development of anxiety, including genetic, neurobiological, and environmental influences.^6^ Common symptoms of anxiety disorder include persistent worry or distress, restlessness, irritability, difficulty concentrating, muscle tension, and sleep disturbances.^7^

Stress, on the other hand, is a state of emotional and physical tension that arises when an individual perceives that environmental demands exceed their ability to cope. Causes of stress can vary from work and financial issues to health and personal relationships. Stress symptoms include anxiety, irritability, fatigue, difficulty concentrating, sleep problems, muscle pain, and digestive disorders.^8^ Stress significantly impacts mental health, as both acute and chronic stress have been linked to increased anxiety and a higher risk of depression.^9^

To assess the severity of depression and anxiety and monitor symptoms over time, various scales are used, such as the Beck Depression and Anxiety Inventories and the Hamilton Scales for Depression and Anxiety.^10,11^ These indices provide objective measures of symptom severity and are widely used in research and clinical practice. Currently, the detection and management of depression and anxiety predominantly relies on self-reporting of symptoms and clinical evaluation.^12^ These limitations can result in delays in recognizing early signs of deterioration, interventions that are often not fully tailored to the individual complexity of each case, which leads to less accurate treatment monitoring and potentially late or inappropriate interventions.^13,14^ However, for stress detection, smartwatches like Fitbit Sense, Garmin Instinct Solar, and the Apple Watch are already available on the market, allowing users to monitor stress levels in real time, offering breathing exercises, guided meditation, and mood tracking.^15,16^

Electronic devices and mobile applications that utilize processing algorithms, physiological signals, and behavioral data offer a new horizon for addressing these challenges. These technologies have the potential to revolutionize mental health diagnosis due to their ability to continuously and objectively collect and analyze data in real time within individuals’ daily lives.^14^ For example, monitoring parameters such as sleep patterns, physical activity, voice tone, and heart rate (HR)—which serve as indirect indicators of an individual's mood—can provide a more comprehensive view of a person's mental well-being.^17,18^ These measures may provide early warning signs of changes in mental health, anticipating the onset of depressive or anxiety episodes, enabling earlier and more personalized interventions.^14,19^

The challenge lies in improving the accuracy and objectivity of diagnostics for highly individual and variable disorders like depression, anxiety, and stress. Furthermore, electronic devices and mobile applications that collect physiological signals and behavioral data offer an opportunity to gather real-time objective information that could transform mental health management. However, their effective use presents challenges related to patient acceptance, privacy, and the clinical interpretation and management of the data produced in clinical practice.^19^

The utility of this literature review lies in the potential to develop non-invasive, accessible diagnostic systems that leverage current technology to identify patterns and trends in physiological signals and behavioral data associated with depression, anxiety, and stress. This approach could enhance clinical care for patients affected by these mental disorders. Additionally, this technological approach may be crucial in addressing resource constraints, providing an affordable and accessible support method for those in need.^20^ These technologies offer a unique opportunity to monitor emotional and mental states through the collection and analysis of physiological signals, behavioral changes, and self-reported symptoms, providing valuable feedback for both professionals and patients.^20,21^

## Methods

This systematic review was conducted between June and September 2024 and considered records from the following electronic databases: PubMed, Google Scholar, IEEE Xplore, Nature, ProQuest, and Science Direct. The aim was to identify relevant scientific literature published in the last decade that addressed the use of physiological and behavioral signals, collected through electronic devices and mobile applications, for the diagnosis or assessment of depression, anxiety, and stress.

**Figure 1.**
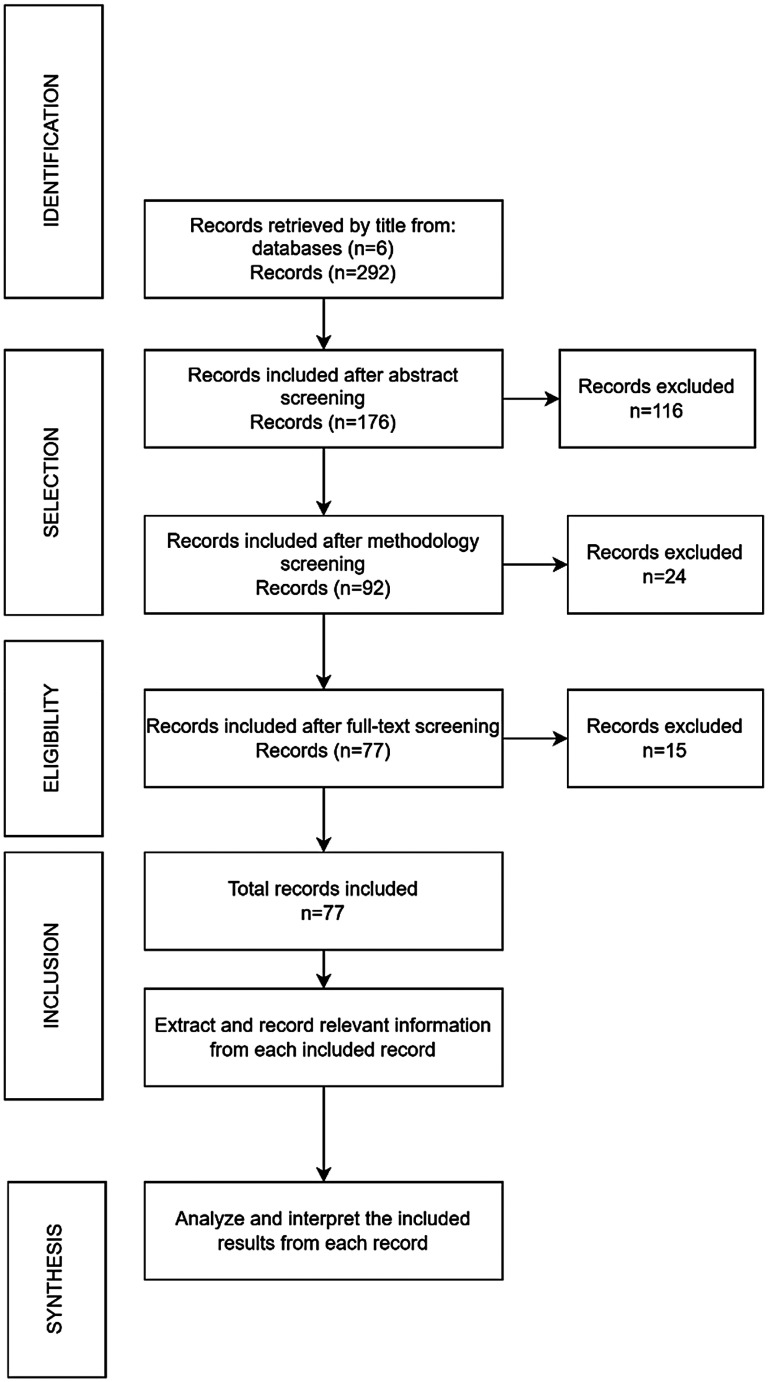
Flowchart of the literature search and results.

This systematic review was conducted in accordance with the PRISMA 2020 guidelines for transparent and reproducible reporting. The review protocol was prospectively registered in the International Prospective Register of Systematic Reviews (PROSPERO) under the ID: CRD420251183329. Data extraction was conducted by a primary reviewer and cross-checked by two independent reviewers to ensure accuracy and consistency. Figure 1 illustrates the multiphase screening workflow.

In the identification stage, a comprehensive search strategy was applied using Boolean operators and the following search equations:

1. (“physiological signals”) AND (“electronic devices” OR “mobile applications”) AND (“depression” OR “anxiety disorder” OR “anxiety” OR “stress”) AND (“diagnosis” OR “assessment”)
2. (“physiological signals” OR “behavioral data”) AND (“electronic devices” OR “mobile applications”) AND (“depression” OR “anxiety disorder” OR “anxiety” OR “stress”) AND (“diagnosis” OR “assessment”)

These queries yielded a total of 292 records, which were initially filtered by title relevance and duplicates removed. One reviewer collected the data reported in this study.

During the selection stage, 176 articles were retained after a thorough review of abstracts. At this point, articles were selected based on their relevance to the topic and the presence of keywords and variables of interest. Studies that did not report any physiological signal, behavioral data, or reference to technological tools were excluded.

In the methodology evaluation, records were examined in greater depth to assess the quality and transparency of the methods. Articles were excluded if they failed to report the specific biomarkers or behavioral indicators measured, the technology or devices employed, or if the methodology was vague or incomplete. After applying these criteria, 92 studies remained.

Finally, in the eligibility stage, 77 peer-reviewed full-text articles were included in the final synthesis. Inclusion was limited to studies that:

- Clearly reported quantitative results on the detection or diagnosis of depression, anxiety, or stress.
- Described the physiological signals measured (e.g. HR, HRV, electrodermal activity (EDA), electroencephalogram (EEG)).
- Specified the mobile technology or electronic device used and its anatomical location on the body (e.g. wrist, chest, head, fingers).
- Reported the population size studied and the targeted mental health condition.
- Provided explicit detail on the data processing methods, including signal preprocessing, statistical analysis, and the algorithms applied.

Articles that lacked information on any of the above aspects were excluded to ensure the methodological rigor and comparability of findings across studies. The comprehensive and systematic approach adopted in this 2024 review aimed to consolidate the current evidence base and identify key trends, gaps, and future directions in the use of wearable and mobile technologies for mental health assessment.

## Results

The results section presents findings from a systematic review of 77 scientific articles that investigate the diagnosis of depression, anxiety, and stress using electronic devices and applications. The analysis covers studies published between 2010 and 2024, emphasizing the growing focus on emerging technologies for identifying and monitoring these mental health conditions. The findings from this review are organized according to the technologies used, device placement, types of variables collected, and algorithms employed across various studies.

### Device placement on the body

Table 1 presents an analysis of the 77 selected studies, with each reference corresponding to a research article investigating the effects of anxiety, depression, or stress using different electronic devices positioned at various locations on the body. The device placements studied include the wrist, chest, head, and fingers. Additionally, a separate category labeled “Other” encompasses devices that do not acquire behavioral and physiological data through direct contact with the body, such as cameras and virtual reality (VR) systems. The studies were categorized based on the disorder under investigation and the placement of the utilized device. Moreover, the average sample size involved in each study was analyzed for each disorder. A total of 31 studies focused on depression, 23 on stress, and 24 on anxiety.

**Table 1.** Classification of studies by device placement on the body.

Placement	Depression	Anxiety	Stress
Wrist	^22–38^	^32,^ ^38–47^	^38,^ ^48–62^
Chest		^63,64^	^48,61,62,65^
Head	^66–70^	^70–74^	^65,74^
Fingers	^75,76^	^75,^ ^77–79^	^80–82^
Other	^83–89^	^85,90^	^16,^ ^91–93^

The placement of electronic devices varies significantly depending on the disorders analyzed. The distribution of placements is as follows: chest (7.8%), head (13%), fingers (10.4%), and wrist (53.2%)—the most common location. Devices that do not require direct body contact, such as cameras and VR diagnostic technologies, accounted for 15.6%. In general, the wrist was the preferred location for diagnosis, with 15 studies focusing on depression, 16 on stress, and 10 on anxiety disorder.

The predominance of wrist-worn devices suggests a preference for comfortable and accessible options, such as smartwatches and fitness bands, which enable the monitoring of multiple physiological variables without interfering with participants’ daily activities. This is particularly beneficial in stress and depression research, where continuous, non-intrusive monitoring is essential.^23,26,27,^^56–58^ On the other hand, the frequent use of head-mounted devices, especially in anxiety and depression studies, is associated with the need to measure specific brain activity parameters.^71,72,74^ In contrast, the low utilization of chest-worn devices may be due to the discomfort they cause, limiting their use in prolonged studies.^59,61,64^

Despite the popularity and convenience of wrist-worn devices, they also have limitations that must be considered. First, while these devices can monitor variables such as HR and physical activity, their accuracy may be compromised by constant wrist movement, leading to potential data inaccuracies. Additionally, the wrist is not an ideal location for measuring deeper physiological parameters, such as core body temperature, which restricts their applicability in studies requiring more specific or complex measurements.

### Physiological signals

Physiological signals are measurable changes in the human body that reflect its physiological state. These signals can be measured continuously over time or as single instances, such as when taking vital signs during a medical consultation. Analyzing these signals enables the study of body function and the detection of patterns associated with health or disease states.^94^ Table 2 presents the classification of the studies analyzed according to the physiological signals measured.

**Table 2.** Classification of studies by the physiological signals measured.

Variable	Depression	Anxiety	Stress
EDA	^16,^ ^26–28^ ^,35,36,44,45,52,63,65,71,77,82,84,100,104^	^26,29,30,45,54,69,77,85,86,93,100^	^16,27,28,33,38,47,65,67,69,76,77,100^
Cardiac measures	^16,^ ^27–31^ ^,35,36,38,45,63–65,67,68,71,74,76,77,79,82,84,88,100,104^	^27,30,31,33,34,46,47,52,63,65,69,72,73,77,85,86,88,90,93^	^16,30,37,46,53,^ ^55–60^ ^,62,67,70,71,78,79,81,84,85,87,89–92^
Temperature	^26,29,37,45,52,65^	^16,64,67,71,77^	^28,30,31,63,70,72,100^
Oxygen saturation	^35,65,68,71,100,104^	^16,28,30,47,52,76^	^27,29,67,77,86,88^
Facial expressions	^16,52,64,71,74,104^	^28,38,65,84,100^	^27,29,30,63,70,77^
EEG	^67–69^	^72,74^	^74^
Eye movement	^45,52,66,91^	^23,27,56,77^	^25,60,65,72^

#### Heart rate

The HR refers to the number of times the heart beats per minute and plays a crucial role in the relationship between depression and anxiety disorder. HR can vary depending on age, physical constitution, physical activity, stress, and other factors. In adults, resting HR ranges between 50 and 100 beats per minute. Studies have shown that individuals with depression exhibit alterations in heart rate variability (HRV), such as low HRV during rest periods and an increased average HR during nighttime. These changes in HRV, indicating a decrease in parasympathetic tone, can precede depression, highlighting a vulnerability to depression.^95,96^ Patients with major depressive disorder have been found to have a HR approximately 10–15 beats per minute higher than healthy patients, suggesting that HR could be a potential clinical biomarker for depression.^97^

Cardiac measures such as HR, HRV, and blood volume pulse (BVP) were used in 74% of the studies. These variables provide detailed insight into physiological responses to stress and anxiety, as well as HR changes associated with depressive episodes.^26,30,52^ HR and HRV are direct indicators of autonomic nervous system activity, and their alteration correlates with intense emotional states such as depression. The findings showed decreased HRV and elevated HR during depressive episodes.^35,63,71,77^ Alterations in BVP and HRV reflect nervous system activation in response to anxiety, providing an objective measure for evaluating emotional state.^29,31,45,54,93^

#### Electroencephalogram

An EEG records the brain's electrical activity, including variations occurring during different sleep stages. It serves as a biomarker for diagnosing depression and anxiety disorders, as several brain circuit-related biomarkers are altered in individuals with these conditions. EEG activity can also be used to classify depression, as different patterns of electrical activity may be associated with distinct subtypes of depression. Additionally, EEG helps explore the relationship between depression and anxiety, as both conditions can be linked to altered brain electrical activity.^28^ Some studies suggest that EEG abnormalities may underlie anxiety disorders in certain patient groups.^98,99^ EEG signals have proven effective in detecting depression, as frontal asymmetry and alpha waves show significant correlation with depressive episodes, providing an objective measure of the associated neural changes.^67^ EEG, employed in 6.5% of the studies, analyzes brain activity and provides information on cognitive responses to stress and anxiety. Using portable EEG in individuals under stress, changes in brain activity were observed during high workload situations.^74^ EEG detects brain activation patterns associated with stress, showing a direct relationship between cognitive activity and stress levels.^16,76,79,85,89^ Brain responses were also observed in high-stress situations, with an increase in high-frequency waves. Brain activity measured with EEG enables the identification of alert and anxious states, supporting its use in diagnosing this condition.^28,79,89,100^

#### Electrodermal activity

EDA is a measure of sympathetic and parasympathetic nervous system activity, which regulates emotional behavior. Anxiety and stress increase sympathetic activity, which is reflected in measurable changes in skin conductance. In individuals with depression, studies have reported reduced EDA amplitude in response to emotional stimuli compared to healthy subjects.^101^ In anxiety, EDA correlates with characteristic symptoms such as excessive sweating, as increased sympathetic activity in anxious states stimulates sweat glands, reducing skin resistance and increasing conductance.^102^

Utilized in 37.6% of articles, EDA is a key indicator of emotional response, as it measures sweat gland activity in reaction to stress and anxiety. EDA sensors were used on individuals in anxiety-provoking and depressive episodes, and the results showed an increase in skin conductance during high anxiety moments.^28,29,45,63,71^ EDA provides an accurate measure of emotional state, and its increase is commonly associated with anxiety episodes, validating its use in diagnosing this condition.^26,30,54,69^ Wrist sensors monitor EDA in participants experiencing stress; EDA is a direct reflection of sweat gland activity, and its increase in stressful situations confirms its effectiveness in stress evaluation.^27,37,38^

#### Temperature

In 23.3% of the studies, temperature monitoring allows for detecting subtle variations in the body that may correlate with stress states. Temperature reflects physiological activity in response to emotions, and its increase during stressful moments confirms its relevance in analyzing this condition.^28,29,31,63^ Temperature sensors were used in stress contexts, with an observed increase in body temperature during emotionally demanding situations. Portable temperature sensors recorded a decrease in temperature during fear situations in anxious individuals. Temperature monitoring enables the capture of physiological responses to stressful and anxious situations, highlighting this variable's sensitivity to emotional changes.^26,37,64,77,85^

#### Oxygen saturation

Oxygen saturation is linked to depression and anxiety disorder in several ways. Stress and anxiety can cause hyperventilation, leading to hypercapnia (increased carbon dioxide concentration) rather than decreased oxygen saturation. Shortness of breath due to anxiety is a sensation of difficulty breathing that occurs when the nervous system is activated in response to a stressful situation. It may be associated with a traumatic or negative experience, a medical condition that makes breathing difficult, or an unhealthy habit that affects respiratory health.^103^

Respiration rates and patterns, present in 23.3% of the studies, are reliable indicators of anxiety and stress states, as they show alterations during these emotional episodes. A chest-worn device was used to monitor respiration in anxious individuals, with data showing an increase in respiratory rate during high anxiety moments.^16,28,47,76^ Respiratory pattern changes during anxiety episodes reflect nervous system activation, consolidating breathing as an anxiety and stress indicator.^27,70,72^ Breathing is significantly altered during depressive episodes, and wearable devices allow for continuous capture of these changes, providing precise information on emotional state.^26,52,65,77^

#### Facial expressions

Used in 22% of articles, capturing facial microexpressions and gestures is effective for identifying emotional responses, especially in diagnosing anxiety. Using cameras to capture micro-expressions, changes in facial expressions indicating increased anxiety were observed. Facial expressions immediately reflect emotions and analyzing them through cameras allows for accurate real-time detection of anxiety.^27,28,84,100^ Reduced facial activity is a characteristic symptom of depression, and facial analysis is a valuable diagnostic tool.^35,64,71,74,104^

#### Eye movement

This variable, represented in 15.6% of studies, allows observation of eye movement patterns associated with emotional responses such as anxiety. Eye movement patterns were analyzed in anxious participants, with avoidance responses observed in anxious situations, and eye tracking allows for precise capture of these patterns.^23,25,27,56^ Reduced visual exploration is common in depression, and eye tracking allows for the non-invasive detection of this symptom.^45,66,91^

### Behavioral variables

Behavioral data can also be captured through electronic devices and mobile applications, which can be analyzed together to identify patterns and trends related to depression, anxiety, and stress. This approach could facilitate early diagnosis and effective monitoring of these conditions. Some relevant data that can be captured include sleep patterns, physical activity, the frequency and duration of social interactions, device usage time, browsing patterns, apps used, voice tone, speech fluency, and mobility patterns. This data can provide important insights.^105,106^

#### Phone usage

Used in 70.1% of the reviewed articles, this category includes app usage, cell activity, and phone calls. Phone activity is a relevant indicator, as changes in usage patterns can reflect emotional states such as anxiety and depression. Mobile applications were used to monitor the phone activity of participants with depression, with a decrease in overall phone usage observed during depressive episodes. App activity and phone calls reflect changes in social behavior and energy levels, making this a relevant marker for detecting depression.^40^

Call logs and app usage records were also analyzed; the frequency and duration of calls are useful variables for observing communication and social interaction levels, which may decrease in disorders such as depression and anxiety. The frequency of phone calls was analyzed, revealing that individuals with depression tend to make fewer calls.^23,40,63,72,73,85,91,108^ Similarly, call duration can reflect a need for social connection as a support mechanism during anxious moments, with an increase in phone usage observed during anxiety episodes, suggesting a potential distraction mechanism.^25,42,51,65,83^

#### Sleep–wake patterns

This category, representing 53.2% of the studies, includes physical activity variables, actigraphy, and sleep duration. Monitoring sleep and physical activity are key components in assessing patterns associated with stress and depression, as disturbances in these areas are common symptoms of these disorders. Wrist-worn devices were used to monitor participants’ sleep duration and physical activity under stress, with results indicating that sleep and physical activity significantly decrease during high-stress episodes. Sleep duration is a clear indicator of stress and anxiety, while actigraphy monitoring captures changes in individuals’ daily routine and energy levels.^26,39,66,92,104^ Reduced physical activity and changes in sleep duration are common symptoms of depression, supporting this category's effectiveness in assessing emotional state (Table 3).^25,51,88^

**Table 3.** Classification of studies by the behavioral variables measured.

Variable	Depression	Anxiety	Stress
Phone usage	^16,^ ^22–24^ ^,33,34,40–42,44,45,48,50,51,56,63,66,67,71,73,82,83,85,86,90,91,107,108^	^16,25,26,29,31,35,40,^ ^42–44^ ^,48,49,51,64,65,72,73,75,76,80,81,83,93,100,104,108^	^16,25,28,38,39,41,43,48,50,57,58,65,77,80,83,87,90,91,107,108^
Sleep–wake patterns	^22–26^ ^,28,41–45,48,51,54,60,66,75,77,80,83,84,88,104^	^22,26,29,30,34,^ ^40–43^ ^,46,49,51,53,66,83,92,107^	^24,25,27,32,39,41,42,45,47,53,72,78,^ ^80–83^ ^,104,108^
Step count	^28,43,45,51,55,62,75,83,85,107^	^25,39,49,66,80,91,100^	^22,24,44,48,50^
Location tracking	^24,43,48,49,63,66,68,76^	^25,26,39,40,44,52,59,74,83,108^	^37,69,75,107^
Ambient light exposure	^36,40,42,50,51,86,108^	^24,41,49,66,70,77,78^	^27,44,48,83,93,100^

#### Step count

Representing 28.5% of the articles, step count provides an indirect measure of daily physical activity, which may significantly decrease in cases of depression. Using a portable step-monitoring device, participants with anxiety were studied, showing a reduction in daily steps during intense anxiety episodes. The reduction in daily physical activity, as measured by steps, reflects a level of withdrawal and low energy during anxious situations.^66,91,100^ Step monitoring offers an indirect measure of activity and motivation, which tends to decrease in people with depression, making it a valuable diagnostic variable.^43,62,83,85^

#### Location tracking

Geographic location tracking was used in 28.5% of the reviewed studies. Geographic mobility is an indicator of routine and social interaction levels, which tend to decrease in individuals with depression and anxiety. GPS-enabled devices allowed geographic mobility tracking, with a reduction in participants’ movement observed. Reduced mobility and confinement to specific locations reflect anxiety symptoms, as people tend to avoid social places during these episodes.^37,39,59,108^ Location and geographic mobility are direct indicators of routine, which tends to diminish during depressive episodes.^24,43,76^

#### Ambient light exposure

This variable was used in 26% of the articles, and monitoring allows for the detection of light exposure levels, which can influence mood and sleep. Using ambient light sensors, light exposure levels were measured in individuals with anxiety, revealing a relationship between low natural light exposure and elevated anxiety symptoms. Ambient light exposure affects mood and sleep quality, making it an important measure for assessing emotional state in individuals with anxiety.^36,41,70,78^ Higher exposure to natural light was found to correlate with a reduction in reported stress levels. Light exposure is a key factor for mood regulation and monitoring can help mitigate the effects of stress by promoting greater exposure.^48,93,100^

### Type of technology

**Figure 2.**
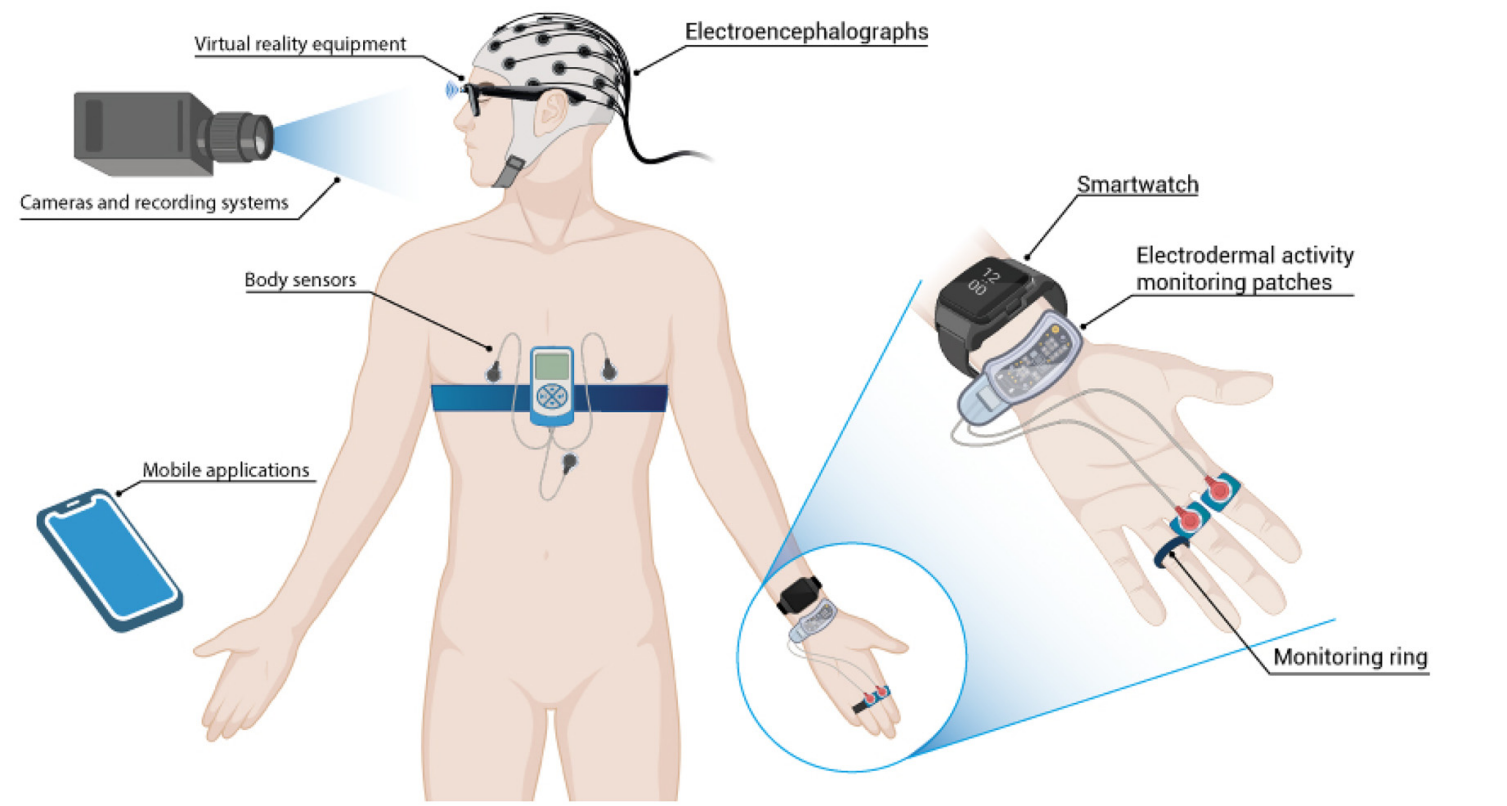
Device placement and technologies used. Created with BioRender.com.

Table 4 groups the devices used for diagnosing depression, anxiety disorder, and stress into different categories. Each of these categories is described below. The body location of these types of technologies is shown in Figure 2.

**Table 4.** Classification of studies by the type of technology used.

Device	Depression	Anxiety	Stress
Smartwatch	^22,23,^ ^25–29^ ^,32,35–38^	^32,38,40,^ ^45–47^	^38,48,49,51,^ ^55–59^ ^,61,62^
Cameras and recording systems	^83,88^	^78^	^82,91^
Electroencephalographs	^67–69^	^72,74^	^74^
Mobile applications	^24,27,35,75,100,104,107^	^75,100,108^	^51,52,100^
Body sensors	^70^	^39,41,42,63,64,70,77,79^	^48,60,65,80,92^
Physical activity bands and rings	^24,75^	^43,71,75^	^53^
Virtual reality equipment	^89^	^39–41^ ^,63,79^	^65,92^

#### Smartwatch

Wearable smartwatch devices were widely used in reviewed studies, representing 33.8% of all analyzed articles. These devices allow the measurement of physiological and behavioral variables such as sleep patterns, EDA, HR, and physical activity, which are relevant indicators for detecting stress, anxiety, and, to a lesser extent, depression. The HR correlates with stress and anxiety, while physical activity is associated with depression. Continuous monitoring with smartwatches enables the detection of fluctuations in physiological responses directly related to these disorders. Smartwatches use a variety of advanced sensors and modules, allowing for precise and continuous monitoring of physiological and behavioral parameters. The reviewed studies show extensive use of technologies such as the PPG sensor, which is essential for measuring the HR and HRV. For example, in the Empatica E4, the PPG sensor detects fluctuations in blood flow correlated with HR changes.^23,25,28,46,59^ Devices like the Garmin Vivosmart 4 also include sensors for measuring EDA, a direct indicator of sympathetic nervous system activation, critical for detecting stress and anxiety.^32,48,57,58^ Accelerometer and gyroscope modules allow the measurement of physical activity and sleep patterns by monitoring device movement and orientation. For example, the Fitbit Flex 2 uses a 3-axis accelerometer to record daily and nightly activity, helping to detect depression-associated changes.^25,28,38^

#### Cameras and recording systems

Camera and recording systems, including devices such as webcams and motion sensors, comprise 6.5% of the articles. These systems were commonly used to capture facial expressions, body movements, and emotion recognition. This type of device shows a high correlation with stress detection (40%) and, to a lesser extent, anxiety (20%). Studies analyzing facial expressions have found that changes in facial behavior are early indicators of anxiety states. Examples include high-definition cameras like the Logitech C1000E, which capture facial micro-expressions and body movements in detail, facilitating the identification of anxiety signs.^78,83^ Combined with image analysis algorithms, these cameras are reported to effectively recognize patterns associated with altered emotional states. Devices like the Kinect use infrared sensors to create 3D maps of body posture, detecting physical tension related to stress.^82^ Pipeline Face2PPG technology estimates HR by analyzing facial skin coloration changes in videos, a non-contact approach for assessing stress.^91^

#### Electroencephalographs

EEG devices, which measure brain electrical activity, represent 6.5% of the studies and were predominantly used for depression detection. The brain activity patterns recorded by EEGs are beneficial for identifying alterations in neuronal rhythms closely associated with depressive episodes. EEG signals are effective in detecting depression, with frontal asymmetry and alpha waves significantly correlating with depressive episodes, providing an objective measure of associated neural changes.^67^ Devices like the Emotiv EPOC and the 16-channel HydroCel Cap capture brain signals via multiple electrodes placed at strategic points on the scalp. In some studies, these devices detect alterations in alpha and beta waves, which are crucial for detecting depression and anxiety.^72,74^ EEGs incorporate amplifiers to capture even the weakest neural signals, which are essential for monitoring frontal asymmetry, and are significant markers in evaluating depressive episodes.^68^ Technologies like EmotivPRO, combined with these devices, enable real-time analysis of brainwave patterns, facilitating the correlation of these signals with stress and anxiety states.^74^

#### Mobile applications

Mobile applications represent 13% of the total reviewed studies. These tools allow for collecting passive data, such as phone usage, location, social interaction, and active data like questionnaire responses. Their versatility and accessibility make them a popular choice for long-term patient monitoring. Patterns of phone usage and decreased social interactions are highlighted as effective predictors of depression. Mobile applications enable continuous, non-invasive tracking of these variables, providing valuable data for longitudinal evaluation.^35,100,104^

Applications like the StudentLife App and PACO App use phone GPS to record movement and location patterns, illustrating how this data helps identify signs of depression and stress based on reduced mobility and avoidance of specific areas.^52,104^ Some applications integrate data from phone accelerometers and gyroscopes to measure physical activity and sleep patterns, emphasizing how these variables are key for depression detection.^75,107^ Monitoring social interactions and speech is also achieved through the phone's microphone, with the AWARE app using this technology to detect changes in call frequency and tone, which may correlate with anxiety.^100,108^

#### Body sensors

Body sensors, such as skin electrical activity patches, electrodes, and HR monitoring devices, are present in 16.9% of the studies. These devices are fundamental in stress detection, as they measure immediate physiological responses like increased skin conductance or HR changes, directly related to stress episodes. EDA and HR monitoring allow for high-precision detection of physiological stress responses, validating these devices’ use in clinical and everyday settings.^39,42,60,80^ Devices like the Shimmer3 and BITalino PLUX use EDA sensors to measure EDA, a critical parameter for detecting stress and anxiety.^41,63,64,92^ ECG sensors, like the Biopac System, measure HR and HRV to evaluate cardiovascular response to stressful stimuli.^77,79,92^

The RespiBAN integrates sensors to monitor respiratory rate, useful in assessing anxiety and stress states, as changes in breathing directly correlate with autonomic nervous system responses in stressful situations.^48,64,80^

#### Physical activity bands and rings

Wearable devices such as physical activity bands and monitoring rings account for 6.5% of the studies. These devices were used to track variables like the HR, sleep, and physical activity, mostly associated with depression and stress, providing an effective tool for monitoring changes in physical activity and sleep. The recorded HR and physical activity correlate with depressive symptoms and stress levels in monitored subjects. The Oura Ring and Microsoft Band 2 use PPG sensors to measure HR, with studies describing how these variables correlate with stress and depression..^53,71,75^ Devices like the Garmin Vivosmart integrate accelerometers to record physical activity and sleep patterns, essential elements for evaluating depression in longitudinal studies.^24,75^

#### VR equipment

VR equipment is present in 10.4% of the studies and was used to elicit emotional reactions and physiological responses in simulated environments. VR equipment was used to assess anxiety, exploring how controlled exposure to simulated environments induces and evaluates emotional responses, allowing precise and controlled anxiety level measurements.^39,40^ Devices like the HTC Vive Pro Eye include sensors to record eye movement and assess attention and reactivity in simulated situations, with reports showing that eye tracking allows real-time anxiety measurement. VR systems can integrate HR and EDA sensors to monitor user responses in controlled environments, assessing physiological stress responses.^65^ The combination of sensors with specialized software, like Unity 3D, allows for the creation of personalized scenarios that elicit specific emotional reactions, facilitating the assessment of anxiety and stress in controlled contexts.^63,92^}

### Algorithms and statistical analysis techniques

In recent years, there has been an increase in the use of technological devices for monitoring physiological signals to diagnose mental disorders such as depression, anxiety, and stress. These devices collect relevant data that, through algorithms and statistical analysis techniques, allow for identifying physiological and behavioral patterns associated with these conditions. This article analyzes the most commonly used algorithms in recent studies, categorizing them based on their application in detecting depression, anxiety, and stress. Studies also include a crucial preprocessing stage to improve data quality before analysis.

Primarily, filters such as the moving average filter were widely used, especially for variables that measure continuous signals like HR, sleep, and physical activity. This filter helps eliminate high-frequency noise in the data, smoothing the signal to provide a clearer representation of changes over time.^24,35,39,56,70,79^ The low-pass filter was commonly used for physiological signals such as electrocardiogram, photoplethysmography, and blood oxygen saturation. This filter allows low frequencies to pass while eliminating high frequencies, which suppresses movement artifacts and environmental noise. This type of filter is essential in analyzing HR or respiratory signals, which may be affected by rapid fluctuations.^52,65,81^ The band-pass filter was used for signals like EEG and EDA. This filter allows only a specific range of frequencies to pass, eliminating both low and high-frequency noise, and is useful for isolating specific frequencies associated with physiological phenomena, such as brain waves in EEG.^63,65,71,99^

Handling missing data is essential, especially in real-time monitoring, where sensor interruptions or device errors can cause data gaps. Imputation techniques, such as mean filling or interpolation, were often used to complete data, ensuring continuity in time-series data.^19,28,50,62,82,107^ Outliers, especially in physiological data like HR or skin conductance, can result from sudden non-representative spikes or sensor artifacts. Techniques like normalization help identify and eliminate these outliers to reduce noise..^32,56,59,87^

**Figure 3.**
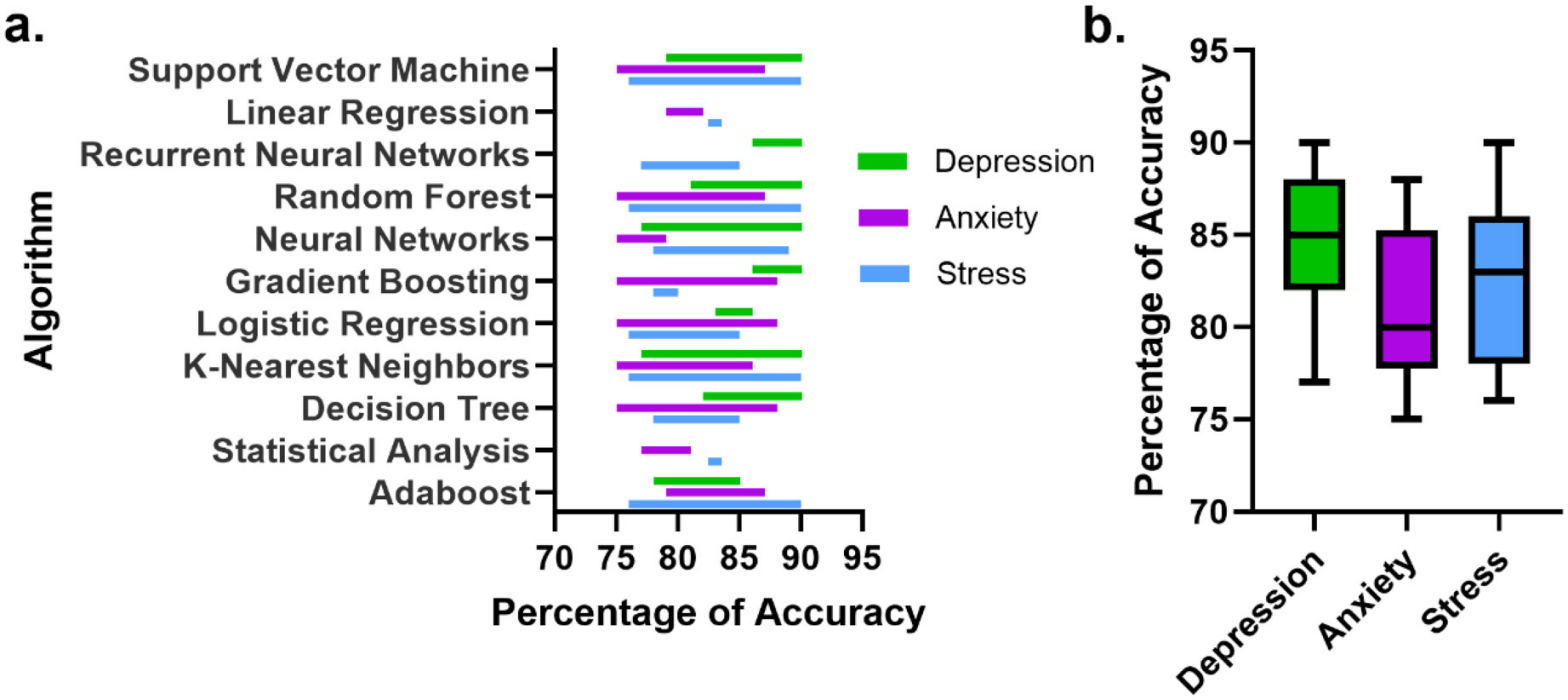
Accuracy performance of algorithms and statistical analysis in detecting depression, anxiety, and stress. (a) Accuracy ranges per algorithm categorized by depression, anxiety, and stress. (b) Boxplot comparison showing distribution of accuracy percentages categorized by depression, anxiety, and stress.

Table 5 summarizes the algorithms and statistical analysis techniques used in the reviewed studies. Figure 3(a) illustrates the accuracy percentages achieved by each algorithm in relation to the three mental health conditions: depression, anxiety, and stress. All algorithms demonstrated accuracy rates above 75%, with depression showing the highest performance (mean accuracy of 85%), while anxiety generally exhibited lower accuracy values, with a mean of 80%.

**Table 5.** Classification of studies by algorithms and statistical analysis techniques.

Algorithm	Depression	Anxiety	Stress
Adaboost	^23,34,35^	^43,74^	^56,58,62,80,82^
Statistical analysis		^39,46^	^81^
Decision tree	^31,66,85,87^	^72,108^	^16,61,93^
k-nearest neighbors	^30,37,66,76,85,88,89^	^41,72,90^	^16,50,55,80,82^
Logistic regression	^26,33,35,85,87^	^44,71,72,108^	^54,61,62^
Gradient boosting	^66,87^	^72,108^	^16,93^
Neural networks	^30,33,34,37,67,70,83,84^	^71,72^	^16,51,^ ^59–62^
Random forest	^26,^ ^29–32^ ^,34–38,84,88,89^	^41,43,44,64,71,72,79^	^16,48,^ ^50–52^ ^,54,56,58,61,62,82,92,93^
Recurrent neural networks	^68,83^		^38,53,91,100^
Linear regression		^40,77^	^65^
Support vector machine	^30,31,37,66,69,76,85,88,89^	^41,44,47,63,64,72,90,108^	^56,58,62,80,82,92,93^

#### Adaboost

In studies related to stress detection, Adaboost showed accuracy above 80%, and for depression detection, results with accuracies up to 85% were reported. The general accuracy range for this algorithm is 76%–90%. The most common metrics were recall, used especially to ensure that subjects with high stress levels were correctly identified—minimizing false negatives—and F1-score, crucial in contexts where classes were imbalanced. Notable variables included HR, EDA, and HRV.^35,62,74^

#### Statistical analysis

Although statistical analyses like the ANOVA and Pearson correlation are not classification algorithms, they were used to explore associations in preliminary studies. Significant associations (*p* < 0.05) were reported between stress and physiological variables such as EDA, respiratory rate, and physical activity.^81^ The *p*-value was key in evaluating these associations, helping identify relevant variables before applying more complex models.^39,46^

#### Decision tree

Decision tree algorithms showed moderate performance, with accuracies ranging from 78% to 90% in detecting depression and stress. This algorithm is useful for interpretable models that are easy to implement in clinical settings.^31,87,93^ The confusion matrix allowed for a detailed evaluation of successes and errors, particularly for adjusting models in detecting anxiety and stress, where physiological signals like body temperature, physical activity, and HRV can be challenging to classify.^61,66^

#### K-nearest neighbors (K-NN)

K-NN was effectively used in studies on anxiety and depression, extracting variables such as the HR, activity level, and body movements, achieving accuracies between 75% and 90%.^85,88,90^ The confusion matrix allowed for a detailed evaluation of the algorithm's performance in correctly classifying subjects with anxiety, measuring both true positives and false negatives.^16,37,72^

#### Logistic regression

Logistic regression was primarily used in studies of depression and anxiety, reaching accuracies between 75% and 88%.^33,85,108^ This classical technique was applied to model the probability of symptom presence based on variables such as physical activity level, respiratory rate, and EDA.^26,44,72,85^ Accuracy was used to evaluate overall model performance, while recall was prioritized in scenarios where precise identification of individuals with anxiety or depression was essential to avoid false negatives.

#### Gradient boosting

This algorithm showed outstanding performance in detecting depression and stress, with accuracies up to 90% and a general range of 78%–90%. It utilized variables such as HR, physical activity, and EDA.^72,93^ Accuracy and AUC-ROC were key metrics; the latter was especially useful for evaluating the model's discriminative ability in datasets with high variability.^87,108^

#### Neural networks

Neural networks, particularly multilayer perceptrons and convolutional neural networks, were used for depression and stress detection.^33,37,59,61^ Studies reported accuracies ranging from 77% to 90%, indicating strong performance in identifying complex physiological patterns such as ECG and EMG signals.^37,59,62^ The F1-score and AUC-ROC were the main evaluation metrics, especially important in classifying depression cases with subtle physiological features.

#### Random forest

Random Forest demonstrated strong performance in detecting depression and stress, achieving accuracies above 85% in most papers within a range of 76%–90%. Known for its ability to handle non-linear and noisy data, it proved effective in classifying physiological signals such as EDA and HR.^32,34,50,56,92^ The F1-score was mainly used to balance precision and recall, particularly in datasets with class imbalance.

#### Recurrent neural networks

Recurrent neural networks (RNNs), such as long short-term memory (LSTM) networks, achieved accuracies above 90%, with a range of 90%–94% in detecting depression and stress.^68,91,100^ Their ability to analyze temporal sequences is key to identifying evolving physiological patterns such as HR, skin conductance, and respiratory rate.^53,83^ AUC-ROC and F1-score were the primary metrics, providing a robust evaluation in high-complexity scenarios.

#### Linear regression

This method was used mainly in exploratory studies of anxiety and stress. While not as accurate as advanced models, linear regression reported accuracies between 80% and 85%.^40^ It was useful for modeling direct relationships between symptoms and physiological variables like HR and respiratory rate. Key metrics included the coefficient of determination (*R*^2^) and the *p*-value, both of which helped measure model fit and identify predictive variables.^65,77^

#### Support vector machine

Support vector machine (SVM) was effectively applied across studies on depression, anxiety, and stress, with accuracies ranging from 75% to 90%.^30,44,66,69,90^ It was particularly effective for high-dimensional datasets, providing optimal hyperplanes for classification. Common metrics were AUC-ROC, F1-score, and accuracy, chosen for their ability to evaluate models on imbalanced data. The algorithm used features such as the HR, EDA, physical activity, and respiratory rate.^37,64,92^

## Discussion

The systematic review focuses on 77 scientific articles investigating the diagnosis of depression, anxiety, and stress through electronic devices and mobile applications. The primary objective of this review was to assess the current state of these technologies by identifying the most commonly used physiological signals and behavioral data, the devices employed, and the applied processing algorithms, while also exploring future research opportunities in this field. The findings highlight a growing interest in the use of electronic devices such as smartwatches, wearable sensors, and mobile applications, which enable the non-invasive and real-time collection of data. Among the most frequently utilized physiological signals for diagnosing depression are HR and alterations in neurophysiology, including alpha waves, which detect alterations associated with depressive states. In terms of behavioral indicators, fragmented sleep and a decrease in daily steps reflect apathy and disinterest.^25,28,^^41–45^^,88,104^ For anxiety diagnosis, EDA and respiratory rate are key indicators of sympathetic and parasympathetic activation,^30,54,76,77,85^ while intensified mobile phone usage may reflect a search for support or distraction.^16,29,100,108^ In the case of stress, relevant signals include body temperature, EDA, and HR, all of which reflect immediate physiological responses.^28,33,^^55–58^^,92^

The technologies used to measure physiological and behavioral signals offer specialized solutions but present limitations in terms of accuracy and applicability. Smartwatches and fitness bands, such as Fitbit and Garmin Vivosmart, are notable for HR monitoring, achieving accuracy rates between 85% and 95%, though they are susceptible to motion artifacts.^32,48,57,58^ Devices like Empatica E4, used for EDA, achieve accuracy levels above 90%, though their comfort level restricts prolonged use.^22,40,48^ Portable EEG devices such as Emotiv EPOC attain accuracy rates between 80% and 92%, but they are highly sensitive to external noise and require controlled environments.^72,74^ On the behavioral analysis side, applications like StudentLife demonstrate an average accuracy of 85% in detecting phone usage patterns and mobility, though they depend on the quality of mobile phone sensors.^52,104^ Cameras such as the Logitech C1000E achieve accuracy rates between 80% and 90% for facial expression analysis but face challenges related to privacy and positioning.^78,83^ VR devices like HTC Vive Pro Eye offer accuracy levels above 90% in experimental settings, though their high cost and technical requirements limit their implementation.^39,40^

The reviewed studies investigating the diagnosis of depression, anxiety, and stress through electronic devices have led to significant advancements but also highlight several areas where further research is needed. A recurring issue is the lack of generalizability of obtained results, as many studies were conducted under controlled conditions with limited population samples, restricting their applicability to real-world scenarios. Future research should focus on conducting studies in everyday environments with a more diverse range of participants, which would allow for model validation in daily-life situations and ensure results are representative of the general population.^43,69,92,97^ Another key limitation is the reliance on isolated physiological variables such as HR, skin conductance, and movement. While these metrics can indicate emotional states, they may not provide a comprehensive picture of an individual's mental state. The absence of a multimodal approach that integrates multiple variables limits the accuracy of detecting complex and differentiated emotions, thereby reducing the effectiveness of current diagnostic algorithms.^55,63^

It was also observed that electronic devices and applications used in these studies present challenges related to comfort and usability. Some devices, such as chest patches or wrist sensors, may be uncomfortable for prolonged use and are perceived as invasive, which could affect participant adherence and limit data collection in longitudinal studies.^64,85^ Another emerging issue is the need for personalized diagnostic models. The development of customized models tailored to each individual's physiological and behavioral variables is identified as a key improvement for increasing diagnostic accuracy. Currently, most models are generalized, which may reduce their ability to accurately detect stress, anxiety, or depression levels in different individuals.^35,90,98^

The integration of additional electronic devices, such as voice sensors or smart glasses equipped with discreet cameras for facial expression analysis, could enhance data quality. The use of these devices in combination with technologies such as smart rings and embedded sensors in clothing could provide a less intrusive approach to physiological and behavioral data collection in daily activities.^43,75^ Additionally, these technologies could measure additional variables such as the body temperature, light exposure, and voice tone, offering a broader spectrum of behavioral data.

Some studies suggest that voice tone should be considered as an emotional state indicator, as variations in tone can signal stress or anxiety levels. This type of variable could be particularly useful in environments such as workplaces or social interactions, where verbal exchanges are frequent, and data acquisition could be facilitated through mobile phone microphones.

Several studies propose that personalizing algorithms to adapt to individual characteristics could improve diagnostic accuracy. Personalized models trained to detect specific user patterns could enhance sensitivity to emotional changes. Furthermore, the development of artificial intelligence-based algorithms that integrate multiple physiological and contextual variables has the potential to significantly enhance accuracy in detecting and differentiating complex emotions. Implementing deep learning models and supervised learning techniques would allow these systems to learn unique user patterns, continuously adapting as individuals interact with the devices. Similarly, applying these algorithms in large-scale population studies could enable the use of advanced data analytics to refine the diagnosis of these conditions, generating personalized physiological and behavioral data clusters based on population characteristics.^37,53,85,108^

Also, the comparative analysis of algorithmic performance (Figure 3(b)) reveals that while depression detection models tend to maintain higher and more consistent accuracy rates, the algorithms used for detecting anxiety and stress show broader variability and lower median performance. This discrepancy highlights inherent challenges in modeling these conditions, potentially due to the more dynamic and context-dependent nature of anxiety and stress-related physiological responses. These findings underscore the need to improve algorithmic precision in these domains by adopting more sophisticated modeling strategies such as temporal deep learning architectures (e.g. LSTM) or ensemble methods that integrate diverse physiological and behavioral variables. Enhancing accuracy in anxiety and stress detection will be essential for advancing real-world applicability and clinical reliability in mental health diagnostics.

Another important limitation that warrants discussion is the challenge of aligning device-generated data with standardized clinical protocols. Many studies do not address how continuous physiological data streams, such as the HR or EDA, can be reliably synchronized with clinical events, psychometric assessments, or therapeutic sessions. In the absence of clear methodologies for data annotation and interpretation, there is a risk of producing clinically irrelevant or misleading results. Furthermore, the heterogeneity of devices and algorithms across studies complicates reproducibility, as each system may employ different signal processing techniques, sampling frequencies, or threshold definitions. Addressing these limitations will require the establishment of harmonized data collection standards, the development of interoperable frameworks, and a re-evaluation of diagnostic criteria to facilitate the integration of these objective metrics into clinical workflows.^20–21^

Despite technological advancements, the integration of these devices into clinical psychology practice presents significant challenges. Current psychological diagnostic protocols rely heavily on standardized clinical interviews, psychometric scales, and self-report instruments, which prioritize subjective assessments over continuous physiological monitoring.^11–14^ These established methods, while validated and widely used, may not align with the dynamic, data-driven insights provided by wearable technologies. Integrating device-generated physiological and behavioral data into existing clinical frameworks demands a re-evaluation of diagnostic criteria, workflow adjustments, and the development of interdisciplinary guidelines that bridge psychology, psychiatry, and biomedical engineering. Moreover, psychological professionals may lack the training to interpret complex biosignals or AI-generated outputs, raising concerns about misinterpretation and overreliance on technology. These barriers underscore the need for robust clinical validation, the development of training protocols for practitioners, and the creation of regulatory standards that ensure ethical, accurate, and clinically meaningful use of these emerging tools in psychological assessment and treatment.^20–21^

The clinical implications of integrating these technologies into routine care are particularly promising. Continuous physiological and behavioral monitoring could enable the early detection of symptom exacerbations, allowing for timely therapeutic interventions and reducing the risk of severe depressive or anxiety episodes. For example, real-time alerts generated by wearable devices could notify healthcare providers of significant physiological deviations, enabling proactive outreach or adjustment of treatment plans. This approach could complement existing therapies, including cognitive behavioral interventions and pharmacological treatments, by providing objective biomarkers of therapeutic response and improving patient adherence through feedback loops. Integrating these tools into clinical practice also has the potential to transform mental health care from a reactive to a preventive model, thereby improving long-term patient outcomes and reducing healthcare costs.

A crucial direction for future research is fostering close collaboration with healthcare professionals to validate these technologies. Clinical feedback is essential to ensure that the developed devices and algorithms can be effectively integrated into medical practice. Such collaboration should focus on adapting devices and algorithms to clinical diagnostic and monitoring protocols, facilitating acceptance among mental health professionals and enabling more precise diagnostic and monitoring approaches. For instance, integrating early warning systems into electronic devices could notify healthcare professionals when a patient exhibits high-risk signs, allowing for timely interventions.

Future research should also prioritize the personalization of diagnostic models, leveraging machine learning techniques capable of adapting to each individual's baseline physiology and behavioral patterns. This could include federated learning approaches that enable decentralized model training while preserving patient privacy. In addition, longitudinal studies conducted in naturalistic settings are essential to evaluate the stability and accuracy of these models over time, particularly across diverse populations. Close collaboration with healthcare professionals is required to validate these technologies and facilitate their integration into clinical diagnostic and monitoring protocols, ensuring acceptance and enabling the development of early-warning systems that allow timely interventions.^43,69,92,97^ Also, future research should work in the integration of advanced technologies, such as embedding sensors in wearable accessories and utilizing flexible electronics and biosensors for continuous, real-time monitoring. For example, smart shirts with embedded sensors could measure HR and respiratory activity,^109,110^ while wristbands equipped with biosensors could detect changes in body temperature or stress levels by analyzing cortisol in sweat.^111–113^ Mobile applications with AI-driven early warning systems could facilitate remote monitoring by generating automated reports for healthcare professionals. Additionally, advanced biosensors such as chemical sensors for cortisol detection or optical sensors for oxygen saturation could expand the range of biological markers associated with these disorders.^114,115^ These technologies are already being explored in devices like flexible electronic patches that monitor skin hydration or smart contact lenses capable of measuring glucose levels in tears.^116,117^ These innovations will require interdisciplinary collaboration to ensure that technologies remain accurate, accessible, and ethically designed, fostering holistic and personalized diagnostic solutions.

## Conclusion

Electronic devices and mobile applications offer a promising approach for diagnosing and monitoring depression, anxiety, and stress due to their ability to record physiological and behavioral data continuously and noninvasively. However, their effective implementation still faces significant challenges regarding accuracy, personalization, and user acceptance. To ensure the optimal integration of these technologies into clinical practice, it is crucial to conduct studies in real-world settings and work closely with healthcare professionals. Such collaboration would enable the development of diagnostic models that are better adapted to the individual characteristics of each patient, facilitating early and personalized interventions.
